# The role of ^18^F-FDG PET/CT in detecting rare post-surgical cardiac metastasis of metaplastic breast carcinoma: a case report

**DOI:** 10.3389/fonc.2025.1521361

**Published:** 2025-04-11

**Authors:** Taiping Liao, Guoxu Fu, Lingxiao Li, Qinlin Qi, Li Li, Yongjun Long

**Affiliations:** Department of Nuclear Medicine, The Third Hospital of Mianyang (Sichuan Mental Health Center), Mianyang, China

**Keywords:** 18F, FDG, PET/CT, metaplastic breast carcinoma, cardiac metastasis

## Abstract

Metaplastic carcinoma of the breast is an extremely rare and highly aggressive malignancy associated with a poor prognosis. Breast spindle cell carcinoma is a subtype of metaplastic carcinoma. We present the case of a 48-year-old woman who was found to have a breast nodule during a routine examination two years ago. Pathological examination following surgical resection confirmed the diagnosis of breast spindle cell carcinoma. One year later, a follow-up CT scan detected a progressively enlarging mass in the left lower lobe of the lung, which was histologically confirmed as metastatic breast spindle cell carcinoma after surgical excision. Recently, the patient developed chest discomfort and severe left thigh pain, prompting an^18^F-FDG PET/CT scan, which revealed metastases to the lung, heart, pleura, and femur. Subsequently, the patient’s condition deteriorated rapidly within a short period. This report highlights the rare imaging findings of cardiac metastasis following surgery for breast spindle cell carcinoma, underscoring the highly aggressive nature of this tumor and the pivotal role of^18^F-FDG PET/CT in the post-operative monitoring of patients with breast spindle cell carcinoma.

## Introduction

Metaplastic breast carcinoma is a rare subtype of breast cancer characterized by squamous or mesenchymal differentiation, which may exhibit spindle cell, chondroid, osseous, or rhabdomyoid differentiation patterns (1). Spindle cell carcinoma, a subtype of metaplastic breast carcinoma, is even rarer, and cardiac metastasis from this tumor is exceptionally uncommon. Here, we report the clinical course and management of a patient with breast spindle cell carcinoma, highlighting the highly aggressive nature of this malignancy and the critical role of ^18^F-FDG PET/CT in post-treatment surveillance.

## Case presentation

**Figure 1:**
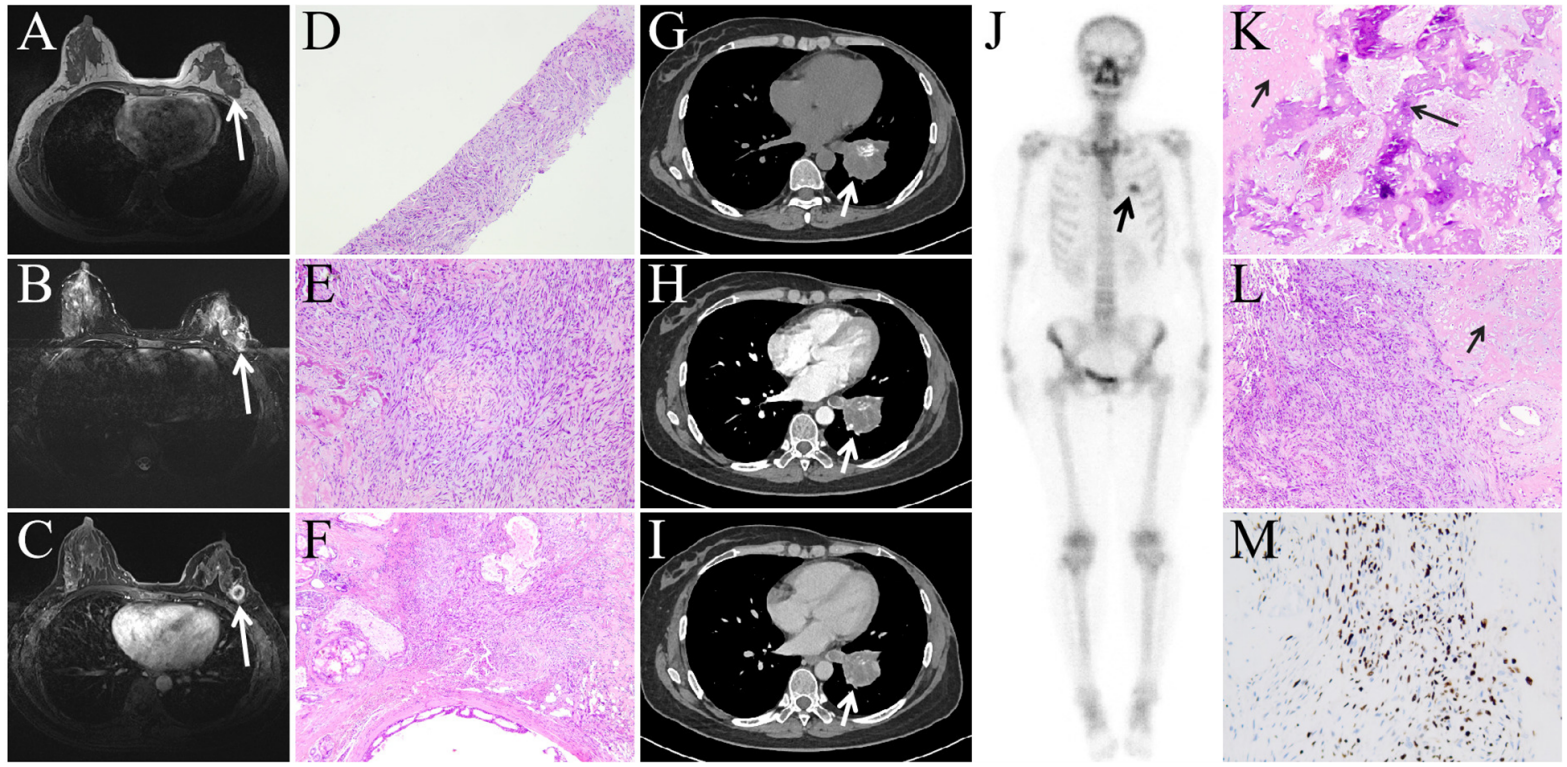
Breast MRI (**A**: T1WI, **B**: T2WI, **C**: contrast-enhanced scan) revealed a nodule in the left upper outer quadrant (long arrow), exhibiting long T1WI and long T2WI signals, with ring enhancement on contrast-enhanced scan. The biopsy result of the breast nodule (**D**: HE, 4×10, **E**: HE, 20×10, **F**: HE, 10×10) showed numerous spindle cells with heterologous components of bone and cartilage. Contrast-enhanced CT examination **(G-I)** revealed a mass in the left lower lobe (short arrow), with mild enhancement on contrast-enhanced scan, patchy high-density areas were also observed within the mass, which demonstrated intense radiotracer uptake on whole-body bone scintigraphy images (**J**, long arrow),suggesting active bone metabolism within the mass, consistent with the presence of bone and cartilage components found in the postoperative pathological images. Figures **K-M** (**K**: HE, 10×10, **L**: HE, 10×10, **M**: DAB, 10×10) represent postoperative pathological images of the left lower lobe lung lesion, which, similar to the breast lesion case results, also revealed heterologous components of bone (**K**, long arrow) and cartilage (**K-L**, short arrow).

A 48-year-old woman presented for a routine physical examination two years ago, during which an ultrasound detected a nodule in the left breast. Contrast-enhanced MRI suggested a high probability of malignancy (**Figures 1A–C**, long arrow). Fine-needle aspiration of the breast nodule was subsequently performed, and pathological analysis identified heterologous elements of bone and cartilage (**Figures 1D–F**), supporting a diagnosis of metaplastic breast carcinoma. The patient underwent a mastectomy, and postoperative pathological examination confirmed spindle cell carcinoma (**Figures 1K–M**). Immunohistochemical staining demonstrated negative expression of ER, PR, and HER2.

The patient subsequently completed six cycles of chemotherapy with Taxol, doxorubicin, and cyclophosphamide. One year later, a follow-up CT scan revealed a mass in the left lower lobe of the lung (**Figures 1G–I**, short arrow), which progressively enlarged over two months, raising concerns for metastatic disease. A whole-body bone scan was performed, which showed no evidence of bone metastases; however, increased radiotracer uptake was observed in the left lower lobe lesion (**Figure 1J**, short arrow). The patient subsequently underwent surgical resection of the left lower lobe lesion, and postoperative pathological examination confirmed metastatic metaplastic breast carcinoma with negative expression of ER, PR, and HER2.

**Figure 2:**
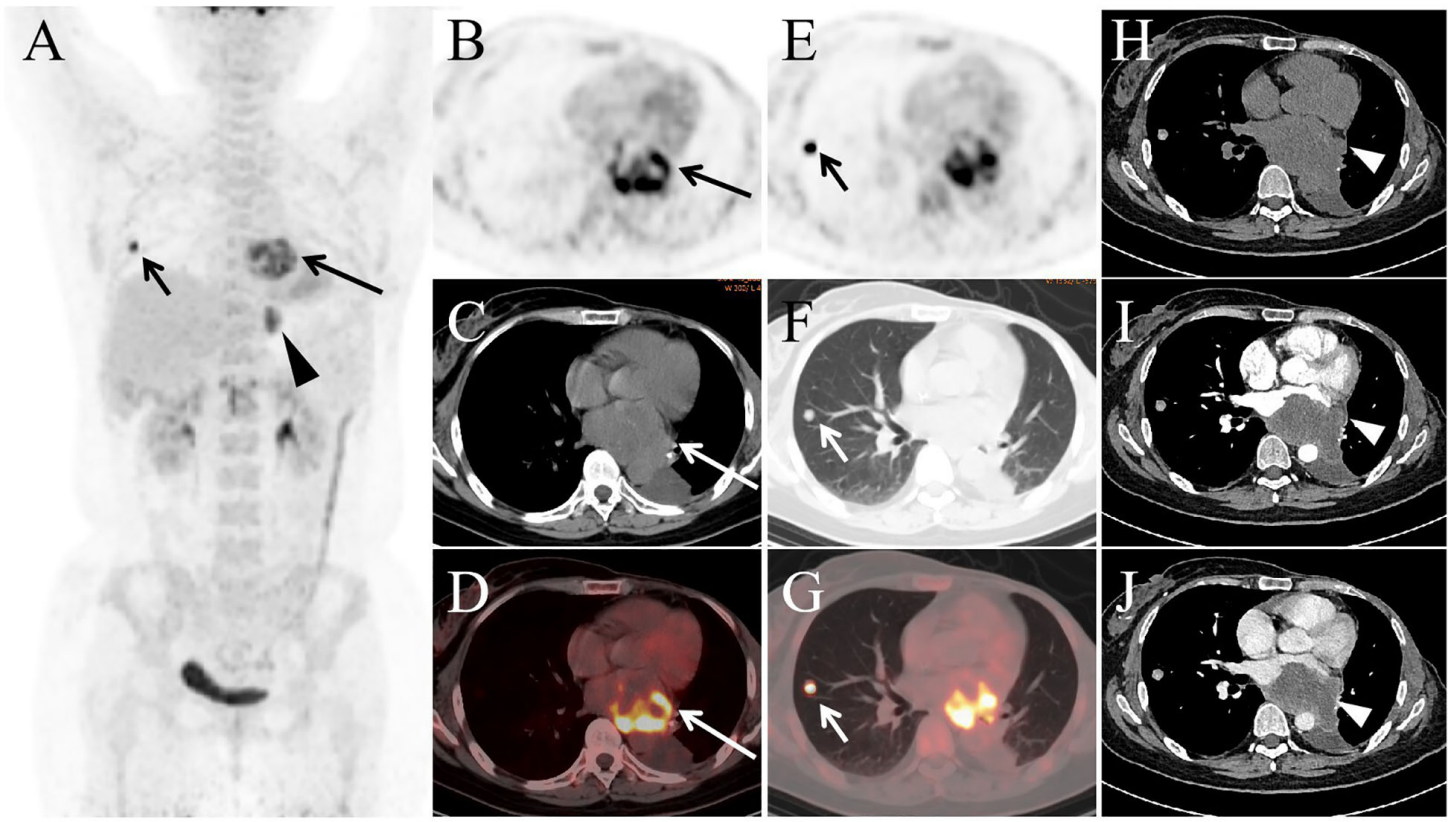
The MIP image **(A)** revealed increased FDG activity in lesions located in the heart (long arrow), right lung (short arrow), and left pleura (arrowhead). Transaxial images (**B**: PET, **C**: CT, **D**: PET/CT) displayed a large soft tissue mass in the left atrial region, exhibiting significantly elevated FDG activity with an SUVmax of 9.5. Additionally, a nodule was observed in the right upper lobe **(E-G)**, showing abnormal FDG uptake with an SUVmax of 10.1. Contrast-enhanced CT **(H-J)** identified mild enhancement of the mass in the left atrium (arrowhead).

Subsequently, the patient was treated with five cycles of Utidelone plus Capecitabine. Utidelone is an analog of epothilone generated by genetically manipulating the polyketide biosynthetic gene cluster in S. cellulosum (2). It is reported that Utidelone demonstrated promising efficacy in the treatment of patients with advanced anthracycline/taxane-refractory metastatic breast cancer (3). One year later, she developed symptoms of fatigue and severe pain in her left thigh. To assess the treatment response and investigate the underlying cause, an ^18^F-FDG PET/CT scan was performed (**Figures 2A–G**). This scan identified nodular soft tissue lesions with abnormal FDG uptake in the right lung (**Figures 2A, E–G**, short arrow) and left pleura (**Figure 2A**, black arrowhead). Notably, an irregular soft tissue mass with markedly elevated FDG uptake was detected in the left atrium (**Figures 2A–D**, long arrow), along with an intramedullary soft tissue density shadow in the upper left femur (not shown in figure) that also demonstrated abnormal FDG uptake. In subsequent follow-up, the patient did not undergo pathological biopsy of the aforementioned lesions, but based on her clinical history and imaging findings, all these lesions were diagnosed as metastatic metaplastic breast carcinoma.

The patient subsequently underwent contrast-enhanced CT (**Figures 2H–J**), which showed mild enhancement of the left atrial mass. She has since received ten sessions of radiation therapy targeting the left femoral lesion, with each session delivering a dose of 3 Gy. Currently, the patient experiences occasional atrial fibrillation, reports poor overall well-being, and is undergoing treatment with Anlotinib and Toripalimab.Anlotinib is a new, orally administered tyrosine kinase inhibitor that targets vascular endothelial growth factor receptor (VEGFR), fibroblast growth factor receptor (FGFR), platelet-derived growth factor receptors (PDGFR), and c-kit (4). Studies have reported that anlotinib is effective in treating advanced metaplastic breast cancer (5, 6).

## Discussion

Metaplastic carcinoma of the breast is an uncommon form of triple-negative carcinoma characterized by the transformation of either a portion or the entirety of its glandular carcinoma component into a non-glandular, or metaplastic, component (7–9). Metaplastic breast carcinoma has a high recurrence rate (10). Spindle cell carcinoma represents approximately <0.3% of invasive breast carcinomas (11). It has a more aggressive biological behavior with increased risk of recurrence and death due to disease compared to triple negative breast cancers (12, 13). The most common site of metastasis for metaplastic breast carcinoma is the lungs (14). Cardiac metastases are relatively rare. Nova-Camacho et al. performed autopsies on 1,294 adult cancer patients and identified 61 cases of cardiac metastases, including three cases of breast cancer (two cases of invasive ductal carcinoma and one case of lobular carcinoma) (15). Taguchi reviewed autopsy studies conducted by other researchers on cancer patients and reported that the incidence of cardiac metastases in malignant tumors ranges from 10% to 15% (16). However, none of these studies documented cases of cardiac metastases from spindle cell carcinoma of the breast. Cardiac metastases from spindle cell carcinoma are exceedingly rare, with reports limited to spindle cell carcinomas originating from the esophagus and larynx (17, 18). Literature has reported the use of ^18^F-FDG PET/CT in diagnosing metaplastic breast carcinoma (19, 20), but there are no documented cases of cardiac metastasis.

We analyzed cases of metastatic spindle cell carcinoma over the past 20 years, as summarized in **Table 1** (17, 18, 21–25). The primary sites of spindle cell carcinoma were most frequently located in the breast. For the treatment of spindle cell carcinoma, monotherapy with pharmacotherapy or radiotherapy was rarely adopted. In cases with limited metastatic spread, a combination of surgical treatment and pharmacotherapy was more commonly selected as the treatment regimen, and the prognosis of most patients was relatively favorable following systematic therapy. However, for patients with widespread metastases before or after treatment, the prognosis was generally poor.

**Table 1:** Summary of the literature in the clinical treatment and prognosis of spindle cell carcinoma with metastases.

Author	Age	Gender	Site of origin	Treatment	Prognosis
Shibata Y (17)	60	Male	Esophagus	Pharmacotherapy (nivolumab)	The patient died rapidly.
Mani N (18)	65	Female	Laryngeal	Surgical and radiotherapy	Five months post-surgery, the tumor metastasized widely, and the patient died.
Nahhat F (21)	40	Female	Breast	Surgical and pharmacotherapy (doxorubicin and cyclophosphamide)	No tumor recurrence at one-year follow-up.
Sakata S (22)	62	Male	Lung	Pharmacotherapy (carboplatin, paclitaxel, and bevacizumab)	The patient maintained the CR for 4 years.He died of leukemia 3 years after completing maintenance therapy.
Toyoda Y (23)	32	Female	Breast	Surgical and pharmacotherapy (FEC followed by triweekly docetaxel)	No tumor recurrence one year after surgery.
Akioka K (24)	60	Male	Kidney	Surgical and radiotherapy	He died of recurrence of renal cancer 3 months after the surgery.
Nagata Y (25)	26	Female	Breast	Surgical and pharmacotherapy (cyclophosphamide, methotrexate and fluorouracil)	The patient developed lung, bone, and brain metastases after treatment and died 2 years post-surgery.
	52	Female	Breast	Surgical and pharmacotherapy (cyclophosphamide, methotrexate and fluorouracil)	No tumor recurrence 7 years after surgery.
	58	Female	Breast	Surgical	No tumor recurrence 5 years after surgery.

In our case, we report a rare occurrence of cardiac metastasis from spindle cell carcinoma of the breast, which is also the first documented case of cardiac metastasis in breast spindle cell carcinoma detected by ^18^F-FDG PET/CT, highlighting the significant role of ^18^F-FDG PET/CT in monitoring metastasis and recurrence in patients with breast spindle cell carcinoma. Unfortunately, despite aggressive treatment, recent enhanced CT scans show that the lung and cardiac lesions continue to enlarge, and the patient has recently developed atrial fibrillation, with new metastatic lesions detected in both kidneys, indicating a potentially poor prognosis.

## Data Availability

The original contributions presented in the study are included in the article/Supplementary Material. Further inquiries can be directed to the corresponding author.
